# Associations of Women’s Health Beliefs and Awareness Regarding Urinary Incontinence and Kegel Exercises with Healthy and Sustainable Lifestyle Behaviors

**DOI:** 10.3390/healthcare14183087

**Published:** 2026-09-19

**Authors:** Ece Kaplan Doğan, Kazım Doğan

**Affiliations:** 1 Obstetrics and Gynecology Nursing, Faculty of Health Sciences, Gaziantep University, Gaziantep 27310, Turkey; 2 Gaziantep Liv Hospital Urology, Istinye University, Istanbul 34396, Turkey; kazim.dogan@istinye.edu.tr

**Keywords:** urinary incontinence, Kegel exercises, health beliefs, sustainable lifestyle

## Abstract

**Background**: Women’s awareness and health beliefs regarding urinary incontinence (UI) and Kegel exercises may influence both symptom management and broader lifestyle behaviors. This study examined associations between women’s health beliefs about and awareness of UI and Kegel exercises and healthy and sustainable lifestyle behaviors. **Methods**: This descriptive, cross-sectional study included 627 women aged ≥18 years. Data were collected using scales: the Health Belief Scale for Urinary Incontinence and Kegel Exercise (UIKE-HBS), the Urinary Incontinence Awareness and Attitude Scale (UIAAS), and the Healthy and Sustainable Lifestyle Scale (HSLS). Relationships were analyzed using bivariate correlations and hierarchical regression. **Results**: UI was reported by 13.7%, and 35.4% had prior Kegel exercise experience. In bivariate analyses, HSLS total score positively correlated with UIKE-HBS Health Motivation, Perceived Benefits, and Self-Efficacy (all *p* < 0.001). In hierarchical regression, covariates explained 5.0% of HSLS variance (R^2^ = 0.050). Adding eleven subscales significantly increased explained variance by 23.6% (ΔR^2^ = 0.236, *p* < 0.001; total R^2^ = 0.286). In the fully adjusted model, UIKE-HBS Health Motivation (β = 0.317, *p* < 0.001), UIAAS Restriction (β = 0.147, *p* = 0.003), UIAAS Accepting Incontinence (β = 0.142, *p* = 0.001) and prior Kegel experience (β = 0.091, *p* = 0.032) emerged as significant independent predictors of HSLS scores. Self-Efficacy and Perceived Benefits were not independently significant in the final model (*p* > 0.05). **Conclusions**: UI was reported by a notable proportion of young women, while engagement in preventive pelvic floor exercises remains limited. Health motivation serves as the primary independent predictor in adjusted models. Interventions targeting health belief constructs specifically enhancing health motivation alongside addressing lifestyle restrictions and acceptance may most effectively foster healthy and sustainable lifestyle behaviors.

## 1. Introduction

Urinary incontinence (UI), defined as the involuntary leakage of urine, represents a common and burdensome lower urinary tract symptom that disproportionately affects adult women [1]. Approximately one-quarter of adult women experience incontinence, including stress, urgency, and mixed subtypes [2]. Demographic and obstetric factors such as increasing age, elevated body mass index, and a history of childbirth are well-established risk factors [3]. Additionally, UI is not merely a physical symptom but is consistently associated with negative impacts on emotional well-being, social participation, and overall quality of life, as affected women often increase hygienic precautions, restrict social activities due to concerns regarding dampness, and experience fear of leakage during intercourse, which frequently leads to intimacy avoidance and relationship strain [4,5].

Pelvic floor muscle training, commonly referred to as Kegel exercises, is widely recognized as a first-line, non-invasive intervention for the prevention and management of UI in women [6,7,8]. Despite its proven effectiveness, adherence to Kegel exercises among women remains suboptimal, largely due to limited awareness, insufficient knowledge, and varying health beliefs regarding UI and preventive behaviors [9]. Although UI is a disease-specific condition, the health beliefs and awareness constructs associated with pelvic floor health such as internal health motivation, bodily self-efficacy, and perceived vulnerability reflect core self-regulatory mechanisms that govern an individual’s broader health orientation. According to holistic health and health-behavior frameworks, individuals who demonstrate strong health motivation and self-efficacy regarding intimate body functions are more likely to exhibit a generalized commitment to overall well-being. Consequently, UI-related beliefs and awareness may share a conceptual pathway with broad lifestyle domains, including physical fitness, emotional health, environmental consciousness, and social awareness, as captured by the Healthy and Sustainable Lifestyle Scale (HSLS) [10].

Despite growing interest in pelvic floor health, the extent to which UI-specific health beliefs and awareness levels relate to comprehensive, sustainable lifestyle behaviors remains insufficiently explored in the literature, particularly among young adult women. Establishing this conceptual rationale addresses an important research gap by clarifying how pelvic floor-specific cognitions align with overarching healthy lifestyle patterns.

Therefore, this study aimed to evaluate the associations of women’s health beliefs and awareness regarding UI and Kegel exercises with their healthy and sustainable lifestyle behaviors.

## 2. Materials and Methods

### 2.1. Study Design

Data were collected online via Google Forms (Google LLC, Mountain View, CA, USA) between 8 January and 28 February 2026. The target population comprised adult women in Turkey aged 18 years and older. A power analysis based on a literature review was conducted to determine required sample size [1,10]. The sample size was calculated using the G*Power software version 3.1.9.7 (Heinrich Heine University Düsseldorf, Düsseldorf, Germany). The minimum number of participants required to detect a significant relationship at a medium-to-large effect size level (Cohen’s d = 0.4166667) between urinary incontinence and awareness among women was determined to be 77 (α = 0.05, 1-β = 0.95). To accommodate complex multivariable linear regression models, minimize potential sampling bias, and enhance subgroup statistical power, a larger sample was recruited, resulting in a final analytical sample of 627 participants. A convenience sample was recruited via open posts on Facebook, Instagram (Meta Platforms, Inc., Menlo Park, CA, USA), and X (X Corp., Bastrop, TX, USA), detailing study objectives, eligibility criteria, estimated completion time (~15 min), voluntariness, and anonymity. Adhering to CHERRIES and STROBE guidelines, the “Limit to 1 response” setting (requiring Google sign-in) was enabled to prevent duplicate submissions without recording personal identifiers, IP addresses, or cookies. All items were set as mandatory, resulting in no missing data among submitted forms. Of the 680 individuals who initiated the survey, 53 abandoned it prior to submission; these unrecorded incomplete entries were excluded. An embedded attention check (“Please select ‘Agree’”) was passed by all 627 respondents who submitted the form, yielding a final analytical sample of 627 participants.

### 2.2. Data Collection Tools

#### 2.2.1. Personal Information Form

The Personal Information Form evaluated participants’ sociodemographic and pelvic floor characteristics. Demographic variables included age, education (literate to postgraduate), employment status, healthcare professional status (yes/no), and marital status. The presence of UI and prior Kegel exercise experience were assessed using single-item binary self-report questions (“yes/no”). Detailed clinical parameters such as UI severity, subtype, duration, parity, and BMI were omitted to ensure a concise format suitable for online dissemination.

#### 2.2.2. Health Belief Scale for Urinary Incontinence and Kegel Exercise (UIKE-HBS)

The UIKE-HBS was developed by Avci and Yildirim (2023) [10]. This 49-item, 5-point Likert scale includes six subscales (without a total score): Perceived Susceptibility, Perceived Severity, Health Motivation, Perceived Benefits, Perceived Barriers, and Self-Efficacy. Subscale scores range as follows: Perceived Susceptibility (9–45), Perceived Severity (14–70), Health Motivation (5–25), Perceived Benefits (7–35), Perceived Barriers (9–45), and Self-Efficacy (5–25). Higher scores indicate increased susceptibility, severity, motivation, perceived benefits, or perceived barriers, depending on the subscale. The Perceived Barriers subscale is negatively oriented, whereas the remaining subscales are positively oriented [10].

#### 2.2.3. Urinary Incontinence Awareness and Attitude Scale (UIAAS)

The UIAAS was developed by Avci et al. (2022) [11]. This 5-point Likert scale comprises five subscales without a total score: Acceptance Barriers, Coping, Health Motivation, Restriction, and Fear. Subscale score ranges are as follows: Acceptance Barriers (8–40), Health Motivation (5–25), Coping (6–30), Restriction (3–15), and Fear (4–20). Higher scores on acceptance barriers indicate greater acceptance of UI as a health problem, while higher scores on Restriction and Fear indicate lower levels of restriction and fear. Lower scores on Health Motivation and Coping indicate better motivation and coping abilities [11].

#### 2.2.4. Healthy and Sustainable Lifestyle Scale (HSLS)

The HSLS was originally developed by Choi and Feinberg and later adapted into Turkish [12,13]. The Turkish version includes 24 items rated on a 5-point Likert scale and comprises six subscales: physical fitness, mental fitness, emotional health, spiritual health, environmentalism, and social awareness. There are no reverse-coded items. Higher scores indicate higher levels of healthy and sustainable lifestyle behaviors [13].

### 2.3. Data Analysis

Statistical analyses were performed using IBM SPSS Statistics for Windows Version 28.0 (IBM Corp., Armonk, NY, USA). Data normality was assessed via the Kolmogorov–Smirnov test and visual diagnostics. The Mann–Whitney U test evaluated group differences across UI status and Kegel experience, while Spearman’s rank correlation evaluated bivariate associations. Hierarchical multivariable linear regression analysis was conducted to identify predictors of HSLS total score. Sociodemographic covariates were entered in Model 1, followed by the simultaneous addition of all eleven subscales from the UIAAS and UIKE-HBS in Model 2. Multicollinearity was ruled out (all VIF values < 10.0), and regression assumptions were verified through residual diagnostics and the Durbin–Watson statistic. Bivariate correlations and group comparisons were evaluated at an unadjusted significance level (*α* = 0.05) within an exploratory framework without adjustment for multiple comparisons; consequently, these bivariate findings should be interpreted cautiously. Hierarchical multivariable linear regression analysis was employed to evaluate independent associations after controlling for covariates and mutual interrelationships among subscales.

### 2.4. Ethics

Written permission for this study was obtained from the Gaziantep University Clinical Research Ethics Committee (date: 7 January 2026; no: 2025/413). Online informed consent was acquired from all participants prior to study inclusion, in accordance with the Declaration of Helsinki (revised in Brazil, 2013).

## 3. Results

The mean age of the participants was 25.93 ± 8.24 years, while the median age was 22 years (IQR: 11; range: 18–60 years) (Table 1).

In exploratory group comparisons, women with UI demonstrated significantly higher sensitivity scores compared with those without incontinence (34.35 ± 5.21 vs. 24.90 ± 6.17, *p* < 0.001). In addition, Coping and Restriction scores were higher among women with UI (*p* = 0.003 and *p* < 0.001, respectively), as were social awareness scores (*p* = 0.001). Participants with prior Kegel exercise experience exhibited higher scores in Perceived Benefits, Self-Efficacy, and all HSLS subscales (all unadjusted *p* < 0.001) (Table 2).

In unadjusted bivariate correlation analyses, the HSLS total score was positively correlated with UIKE-HBS Health Motivation (r = 0.468, *p* < 0.001), Perceived Benefits of Kegel exercises (r = 0.364, *p* < 0.001), and Self-Efficacy (r = 0.317, *p* < 0.001). Positive associations were also observed between HSLS total score and UIAAS acceptance of UI as a health condition (r = 0.320, *p* < 0.001), as well as UIAAS Restriction (r = 0.155, *p* < 0.001). Conversely, the HSLS total score showed significant negative correlations with UIAAS Health Motivation (r = −0.395, *p* < 0.001), Coping with UI (r = −0.129, *p* = 0.001), and Fear of UI (r = −0.093, *p* = 0.020) (Table 3).

UIAAS Accepting Incontinence as a health condition was positively correlated with UIKE-HBS Health Motivation (r = 0.262, *p* < 0.001), Perceived Benefits of Kegel Exercises (r = 0.348, *p* < 0.001), Perceived Barriers to Kegel Exercises (r = 0.232, *p* < 0.001), and Self-Efficacy (r = 0.217, *p* < 0.001). In contrast, UIAAS Health Motivation correlated negatively with UIKE-HBS Health Motivation (r = −0.353, *p* < 0.001), Perceived Benefits (r = −0.446, *p* < 0.001), Perceived Barriers (r = −0.307, *p* < 0.001), and Self-Efficacy (r = −0.231, *p* < 0.001). Additionally, UIAAS Restriction correlated negatively with UIKE-HBS Perceived Severity (r = −0.398, *p* < 0.001) but positively with UIKE-HBS Perceived Barriers (r = 0.212, *p* < 0.001) and Self-Efficacy (r = 0.104, *p* = 0.009). UIAAS Fear of UI was negatively correlated with UIKE-HBS Perceived Susceptibility (r = −0.109, *p* = 0.006), Perceived Severity (r = −0.289, *p* < 0.001), Health Motivation (r = −0.172, *p* < 0.001), and Perceived Benefits (r = −0.101, *p* = 0.011), while showing a weak positive correlation with UIKE-HBS Perceived Barriers (r = 0.088, *p* = 0.028) (Table 3).

A hierarchical multivariable linear regression analysis was conducted to determine the independent predictors of the HSLS total scores after adjusting for covariates (Table 4). In Model 1, the 12 sociodemographic covariates accounted for 5.0% of the variance in the HSLS total scores (R^2^ = 0.050, Adjusted R^2^ = 0.032, F(12, 614) = 2.704, *p* = 0.001). Prior Kegel exercise experience was the only significant positive predictor in Model 1 (B = 5.318, SE = 1.241, β = 0.177, *p* < 0.001). In Model 2, the simultaneous addition of the eleven subscales from the UIAAS and UIKE-HBS significantly improved the model fit, explaining an additional 23.6% of the variance (ΔR^2^= 0.236; ΔF (11.603) = 18,076, *p* < 0.001). The fully adjusted model accounted for 28.6% of the total variance in the HSLS scores (R^2^= 0.286, Adjusted R^2^= 0.258, F(23, 603) = 10.487, *p* < 0.001; Durbin–Watson = 2.063). In this fully adjusted model, UIKE-HBS Health Motivation emerged as the strongest independent predictor (β = 0.317, B = 1.291, SE = 0.162, *p* < 0.001), followed by UIAAS Restriction (β = 0.147, B = 0.748, SE = 0.250, *p* = 0.003) and UIAAS Accepting Incontinence (β = 0.142, B = 0.359, SE = 0.106, *p* = 0.001). Additionally, prior Kegel exercise experience maintained its positive independent contribution (β = 0.091, B = 2.717, SE = 1.265, *p* = 0.032). Notably, subscales that were correlated with the HSLS in bivariate steps such as Self-Efficacy and Perceived Benefits did not retain independent statistical significance in the fully adjusted model (*p* > 0.05).

## 4. Discussion

UI and limited engagement in preventive pelvic floor practices are increasingly recognized as clinically relevant yet underappreciated issues even among young adult women, challenging the perception that these conditions are confined to later life. While our prevalence estimate (13.7%) aligns with studies reporting 11–13% in young women (stress UI being most frequent), comparisons should be made cautiously. A primary consideration when interpreting our findings is the highly selective nature of the study population. Recruitment was conducted via convenience sampling on social media, resulting in a sample predominantly composed of young women (median age: 22 years), 92.0% of whom were university graduates or undergraduates and 61.9% were healthcare workers or healthcare students. These demographic characteristics inherently introduce selection bias and substantially limit the generalizability of the findings to the broader population of women. Individuals with higher education levels and healthcare backgrounds generally possess greater health literacy, higher baseline health awareness, and different health-seeking behaviors compared with the general public. Consequently, the high proportion of healthcare workers and university-educated participants may have inflated awareness levels, positively skewed health belief scores and contributed to higher overall healthy lifestyle (HSLS) scores. Therefore, while younger adult women clearly contribute to the UI burden supporting evidence from focused analyses in early adulthood [14,15]. Our findings regarding awareness, health beliefs, and lifestyle interactions must be interpreted within the context of this health-literate and educated population.

Parallel to UI prevalence, awareness and engagement in pelvic floor muscle training remain suboptimal among younger women. Studies consistently show a gap between knowledge and practice. In a survey of 896 women, although most demonstrated moderate knowledge (80.1%) and positive attitudes (77.3%), 87.2% did not actively practice pelvic floor exercises [16]. Similarly, Ismail et al. reported that while 54.9% of women were aware of these exercises, only 20.5% engaged in regular practice [17]. These findings are consistent with our results, where 35.4% reported prior Kegel experience. Notably, even in our sample with a high representation of healthcare workers, nearly two-thirds of participants had never performed Kegel exercises, indicating that high general health awareness or a medical background does not automatically translate into preventive pelvic floor behaviors. A plausible explanation for this disparity is the potential normalization of mild, non-bothering symptoms among young women and a lack of routine emphasis on pelvic floor health during early clinical encounters.

Coyne et al. reported that women with UI were more likely to adopt compensatory strategies such as fluid control and activity restriction, with significantly higher Coping scores [18]. Consistently, Pizzol et al. found that UI was associated with increased restriction of social and physical activities [19]. In line with these findings, women with UI in our study had higher Perceived Susceptibility, Coping and Restriction scores and social awareness. Despite the relatively low prevalence (13.7%), these results indicate that even mild UI can influence behavioral and cognitive responses, which may tentatively be attributed to heightened concern about potential social consequences [20].

Engagement in pelvic floor muscle training is associated with improved self-efficacy and health-related outcomes. Dumoulin et al. reported that structured training significantly enhanced self-efficacy and perceived benefits [21], while Asklund et al. showed that adherence to eHealth-based programs improved both self-efficacy and quality of life [22]. Additionally, women performing pelvic floor exercises demonstrated higher levels of overall health-promoting behaviors, including physical, emotional, and social domains [23]. Consistent with these findings, participants with prior Kegel exercise experience in our study had higher Perceived Benefits, Self-Efficacy, and HSLS scores. However, given the cross-sectional design of our study, these group differences must be interpreted with caution. It is highly plausible that a self-selection mechanism is at play, wherein women who are inherently more health-conscious, highly motivated, or possess greater baseline self-efficacy are naturally more inclined to learn about and engage in Kegel exercises in the first place. Therefore, prior Kegel experience may serve as an indicator of a broader pre-existing health-seeking orientation rather than being the sole direct cause of higher health beliefs and lifestyle scores. It should be noted that pelvic floor muscle training represents only one component of conservative UI management, and bladder training may also play an important role, particularly in women with urgency or mixed UI.

Growing evidence suggests that UI–related health beliefs are closely linked to broader health behaviors and psychosocial functioning. Melville et al., in a study of 1160 women, demonstrated that stronger perceived severity and symptom appraisal were significantly associated with increased health-oriented behaviors and care-seeking tendencies [24]. Supportively, our study revealed moderate positive correlations between HSLS total score and UIKE-HBS Health Motivation, Perceived Benefits, and Self-Efficacy. Conversely, negative correlations between HSLS and UIAAS Health Motivation, Coping and Fear suggest a potential pattern wherein symptom-focused emotional or avoidant responses inversely correspond with broader health-promoting lifestyle engagement.

Inter-construct evaluations further illustrate the nuanced relationships between awareness and health beliefs. In our study, acceptance of UI as a health condition was positively correlated with UIKE-HBS Health Motivation, Perceived Benefits, Perceived Barriers, and Self-Efficacy. One possible interpretation of the positive association with Perceived Barriers is that cognitive acknowledgment of UI may increase attentiveness to potential obstacles in symptom management, rather than reflecting purely negative appraisal. Conversely, UIAAS Health Motivation negatively correlated with UIKE-HBS Health Motivation, Perceived Benefits, Barriers, and Self-Efficacy, highlighting conceptual distinctions between awareness and belief constructs. Furthermore, the observed negative correlations between fear and multiple belief constructs may reflect a cognitive pattern where fear attenuates adaptive motivational processes. Although interventional evidence by Öz Yıldırım et al. [25] shows that structured belief-based education can restructure cognitive appraisal and enhance self-efficacy, our cross-sectional correlations remain exploratory and require longitudinal validation.

Within our fully adjusted hierarchical framework, controlling for key sociodemographic covariates (Model 1, R^2^ = 0.050) confirmed that the integrated set of health belief and awareness subscales accounted for a substantial increment in healthy lifestyle behaviors (ΔR^2^= 0.236, *p* < 0.001, total model R^2^= 0.286). When evaluating all eleven subscales simultaneously in the fully adjusted model, UIKE-HBS Health Motivation emerged as the single strongest positive predictor of HSLS scores (β = 0.317, *p* < 0.001), underscoring that intrinsic health-seeking drive is the primary cognitive engine aligning urinary health attitudes with broader sustainable lifestyle habits. Additionally, UIAAS Restriction (β = 0.147, *p* = 0.003) and UIAAS Acceptance (β = 0.142, *p* = 0.001) maintained independent positive associations, suggesting that both cognitive acknowledgment of UI and awareness of its daily behavioral impacts independently encourage broader lifestyle adaptations. Notably, prior Kegel exercise experience retained its significant positive contribution even after adjusting for all subscales (β = 0.091, *p* = 0.032), serving as an indicator of an active baseline health-seeking orientation. Crucially, subscales that demonstrated significant bivariate correlations with HSLS such as UIKE-HBS Self-Efficacy and Perceived Benefits did not retain independent statistical significance in the fully adjusted multivariable model (*p* > 0.05). This attenuation indicates that the variance initially shared between these broad cognitive beliefs and health behaviors is absorbed by the more proximal, intrinsic drive represented by Health Motivation, as well as specific condition-awareness dimensions. While these statistical associations indicate that internal motivation and targeted awareness align closely with a general health orientation, interpretations regarding deeper causal mechanisms extend beyond our cross-sectional data. These findings should therefore be viewed as reflecting broad behavioral orientations rather than direct causal predictors of UI management.

## 5. Study Limitations

This study has several limitations. Its cross-sectional design precludes causal inference, and self-reported data may be subject to reporting bias. In particular, the observed differences between participants with and without prior Kegel exercise experience must be interpreted with caution, as women who are inherently more health-conscious, motivated, or self-efficacious may simply be more likely to adopt Kegel exercises initially (self-selection bias). Furthermore, a sample predominantly comprising young, highly educated women limits generalizability to the broader female population. Additionally, specific urinary incontinence (UI) subtypes, conservative management behaviors (e.g., bladder or supervised pelvic floor muscle training), and risk factors relevant to younger women (e.g., intensive physical training) were not evaluated. Using a broad lifestyle scale (HSLS) rather than a UI-specific behavioral instrument means observed associations may reflect general health orientation rather than direct UI management. Consequently, findings should be considered hypothesis-generating. The presence of UI and prior Kegel exercise experience were determined using single-item self-report binary questions rather than clinical diagnostic examinations or validated UI severity instruments. Additionally, clinical characteristics such as UI subtype, duration, parity, and BMI were not recorded, which limits detailed subgroup stratification. Furthermore, although all variance inflation factors (VIFs) remained below the conservative threshold of 10.0 in the fully adjusted regression model, moderate variance inflation was noted for specific educational categories (high school and university education), likely reflecting the uneven distribution of education levels in our sample. Finally, bivariate group comparisons and correlation analyses were conducted without post hoc corrections for multiple testing within an exploratory design, which may increase the potential for Type I error. Accordingly, bivariate associations should be interpreted with caution, emphasizing the fully adjusted multivariable models.

## 6. Future Directions

Future research should be conducted with larger, population-based samples encompassing diverse age groups, educational levels, and socioeconomic backgrounds to enhance the generalizability of the findings. Additionally, longitudinal and interventional study designs are needed to investigate the causal relationship of health belief- and self-efficacy-based educational programs on long-term lifestyle modifications and pelvic floor health. Finally, separately evaluating specific urinary incontinence subtypes (e.g., stress, urgency, mixed) and tailored conservative management strategies such as supervised pelvic floor muscle training or bladder training will help refine targeted clinical practices and preventive public health strategies.

## 7. Conclusions

In this young, highly educated female sample, UI was notably prevalent, whereas preventive pelvic floor muscle training remained low despite moderate awareness. Bivariate analyses initially indicated that constructs such as perceived benefits, self-efficacy, and general awareness were positively associated with lifestyle outcomes. However, in our fully adjusted hierarchical regression model where sociodemographic covariates accounted for 5.0% of lifestyle variance and the integration of urinary health belief and awareness constructs explained a total of 28.6%, these initial bivariate associations did not all translate into independent effects. Crucially, intrinsic health motivation emerged as the primary independent predictor of healthy and sustainable lifestyle behaviors, alongside symptom restriction, incontinence acceptance, and prior Kegel practice, whereas self-efficacy and broad awareness lost statistical significance. Rather than assuming direct interventional outcomes from cross-sectional data, these observational findings demonstrate that health promotion initiatives should look beyond surface-level awareness or isolated self-efficacy to target core internal health motivation. These findings warrant confirmation in larger, representative population-based studies using prospective or longitudinal designs.

## Figures and Tables

**Table 1:** Descriptive characteristics of the study population.

*Variable*	Category	*n*	%
***Age (years)***	Mean ± SD	25.93 ± 8.24	–
***Educational Level***	Primary school graduate	6	1.0
	Middle school graduate	6	1.0
	High school graduate	34	5.4
	University graduate or above	577	92.0
	Literate	4	0.6
***Employment Status***	Working	161	25.7
	Studying	384	61.2
	Housekeeping	77	12.3
	Retired	5	0.8
***Healthcare Worker Status***	Yes	388	61.9
	No	239	38.1
***Marital Status***	Married	212	33.8
	Single	415	66.2
***Urinary Incontinence***	Yes	86	13.7
	No	541	86.3
***Previous Kegel Exercise Experience***	Yes	222	35.4
	No	405	64.6

**Table 2:** Comparison of scale and subscale scores according to urinary incontinence status and history of Kegel exercise.

*Scale/Subscale*	UI Absent (*n* = 541) (Mean ± SD)	UI Present (*n* = 86) (Mean ± SD)	*p*-Value	No Kegel Exercise (*n* = 405) (Mean ± SD)	Kegel Exercise (*n* = 222) (Mean ± SD)	*p*-Value
***UIKE-HBS*** *—Perceived Susceptibility*	24.90 ± 6.17	34.35 ± 5.21	<0.001 *	26.02 ± 7.11	26.52 ± 6.40	0.399
*Perceived Severity*	45.66 ± 9.96	48.20 ± 13.22	0.018 *	46.23 ± 10.40	45.61 ± 10.67	0.744
*Health Motivation*	17.32 ± 3.54	17.29 ± 3.46	0.836	17.04 ± 3.65	17.82 ± 3.23	0.005 *
*Perceived Benefit*	27.21 ± 4.92	27.15 ± 5.44	0.984	26.22 ± 4.80	29.00 ± 4.84	<0.001 *
*Perceived Barrier*	29.68 ± 6.43	28.55 ± 6.33	0.081	27.53 ± 5.45	33.16 ± 6.48	<0.001 *
*Self-Efficacy*	16.73 ± 3.75	16.55 ± 4.58	0.544	15.38 ± 3.42	19.11 ± 3.47	<0.001 *
***UIAAS*** *—Acceptance Barriers*	29.98 ± 5.70	30.56 ± 5.45	0.638	29.58 ± 5.45	30.93 ± 5.96	0.001 *
*Health Motivation*	10.11 ± 3.97	9.42 ± 3.47	0.262	10.53 ± 4.02	9.08 ± 3.53	<0.001 *
*Coping*	16.92 ± 4.96	18.35 ± 4.48	0.003 *	16.86 ± 4.72	17.56 ± 5.24	0.158
*Restriction*	9.53 ± 2.76	10.80 ± 2.97	<0.001 *	9.51 ± 2.69	10.06 ± 3.00	0.029 *
*Fear*	11.11 ± 3.40	11.94 ± 4.31	0.104	11.02 ± 3.37	11.60 ± 3.81	0.175
***HSLS*** *—Physical fitness*	13.82 ± 3.21	13.58 ± 3.51	0.813	13.33 ± 3.29	14.63 ± 3.00	<0.001 *
*Mental well-being*	10.48 ± 2.52	10.28 ± 2.98	0.996	10.20 ± 2.62	10.91 ± 2.46	0.001 *
*Emotional health*	10.69 ± 2.48	10.81 ± 2.78	0.542	10.47 ± 2.54	11.15 ± 2.44	0.001 *
*Spiritual health*	10.74 ± 2.34	10.81 ± 2.59	0.743	10.51 ± 2.37	11.18 ± 2.33	<0.001 *
*Environmentalism*	28.36 ± 5.12	28.43 ± 5.99	0.261	27.82 ± 5.17	29.37 ± 5.24	<0.001 *
*Social awareness*	11.03 ± 2.14	11.77 ± 2.09	0.001 *	10.86 ± 2.17	11.62 ± 2.02	<0.001 *
*Total score*	85.12 ± 14.18	85.69 ± 15.48	0.236	83.19 ± 14.41	88.84 ± 13.54	<0.001 *

Group comparisons were performed using the Mann–Whitney U test. * A *p*-value < 0.05 was considered statistically significant. **UIKE-HBS**: Urinary Incontinence and Kegel Exercise Health Belief Scale. **UIAAS**: Incontinence Awareness and Attitude Scale. **HSLS**: Healthy and Sustainable Lifestyle Scale.

**Table 3:** Spearman correlations between HSLS total/UIKE-HBS and UIAAS subscale scores.

UIKE-HBS Subscale	HSLS Total	UIAAS Acceptance	UIAAS Health Motivation	UIAAS Coping	UIAAS Restriction	UIAAS Fear
***HSLS*** ***Total Score*** *r (p-value)*	-	0.320 (<0.001) *	−0.395 (<0.001) *	−0.129 (0.001) *	0.155 (<0.001) *	−0.093 (0.020) *
***UIKE-HBS*** ***Perceived Susceptibility*** *r (p-value)*	−0.010 (0.803)	0.056 (0.158)	−0.007 (0.858)	0.094 (0.018) *	−0.025 (0.531)	−0.109 (0.006) *
***UIKE-HBS Perceived Severity*** *r (p-value)*	−0.038 (0.348)	0.054 (0.181)	−0.047 (0.237)	−0.011 (0.784)	−0.398 (<0.001) *	−0.289 (<0.001) *
***UIKE-HBS*** ***Health Motivation*** *r (p-value)*	0.468 (<0.001) *	0.262 (<0.001) *	−0.353 (<0.001) *	−0.134 (0.001) *	0.021 (0.602)	−0.172 (<0.001) *
***UIKE-HBS Perceived*** ***Benefits*** *r (p-value)*	0.364 (<0.001) *	0.348 (<0.001) *	−0.446 (<0.001) *	−0.108 (0.007) *	0.071 (0.075)	−0.101 (0.011) *
***UIKE-HBS Perceived Barriers*** *r (p-value)*	0.233 (<0.001) *	0.232 (<0.001) *	−0.307 (<0.001) *	0.059 (0.141)	0.212 (<0.001) *	0.088 (0.028) *
***UIKE-HBS*** ***Self-Efficacy*** *r (p-value)*	0.317 (<0.001) *	0.217 (<0.001) *	−0.231 (<0.001) *	−0.048 (0.231)	0.104 (0.009) *	0.007 (0.865)

* Spearman’s rank correlation was used. *p* < 0.05 was considered statistically significant.

**Table 4:** Hierarchical regression model predicting HSLS total score.

Variable	Model 1 (Covariates)		Model 2 (Fully Adjusted: Covariates + 11 Subscales)		Multicollinearity Diagnostics (Model 2)	
	B (SE)	β	B (SE)	β	Tolerance	VIF
(Constant)	82.549 (8.432)	—	44.577 (9.876)	—	—	—
Age	0.155 (0.128)	0.089	0.133 (0.116)	0.077	0.267	3.743
Middle school graduate ^a^	3.675 (8.224)	0.025	1.757 (7.224)	0.012	0.493	2.030
High school graduate ^a^	−5.606 (6.395)	−0.089	−4.614 (5.646)	−0.073	0.149	6.709
Literate ^a^	−13.894 (9.330)	−0.077	−10.492 (8.230)	−0.058	0.567	1.762
University graduate ^a^	−4.164 (6.170)	−0.079	−3.872 (5.426)	−0.073	0.113	8.868
Married ^c^	−1.015 (2.245)	−0.033	−2.101 (1.981)	−0.069	0.277	3.606
Employment status: Student ^b^	−0.303 (2.297)	−0.010	1.086 (2.088)	0.037	0.235	4.248
Employment status: Housewife ^b^	1.020 (2.282)	0.023	1.499 (2.033)	0.034	0.547	1.827
Employment status: Retired ^b^	−5.926 (7.073)	−0.037	−4.122 (6243)	−0.026	0.790	1.265
Do you have urinary incontinence?	−0.225 (1.795)	−0.005	0.878 (1.756)	0.021	0.668	1.497
Have you ever performed Kegel exercises?	5.318 (1.241)	0.177 ***	2.717 (1.265)	0.091 *	0.665	1.503
Healthcare professional status	0.645 (1.389)	0.022	−0.485 (1.246)	−0.016	0.665	1.503
UIAAS—Accepting Incontinence	—	—	0.359 (0.106)	0.142 **	0.674	1.485
UIAAS—Health Motivation	—	—	−0.117 (0.166)	−0.032	0.579	1.727
UIAAS—Coping	—	—	−0.125 (0.118)	−0.043	0.724	1.381
UIAAS—Restriction	—	—	0.748 (0.250)	0.147 **	0.490	2.041
UIAAS—Fear	—	—	−0.145 (0.192)	−0.036	0.528	1.896
UIKE-HBS—Perceived Susceptibility	—	—	−0.107 (0.092)	−0.051	0.606	1.650
UIKE-HBS—Perceived Severity	—	—	−0.070 (0.059)	−0.051	0.640	1.561
UIKE-HBS—Health Motivation	—	—	1.291 (0.162)	0.317 ***	0.750	1.333
UIKE-HBS—Perceived Benefits	—	—	0.200 (0.131)	0.070	0.571	1.751
UIKE-HBS—Perceived Barriers	—	—	−0.057 (0.098)	−0.026	0.619	1.616
UIKE-HBS—Self-Efficacy	—	—	0.290 (0.168)	0.078	0.575	1.740
R^2^	0.050		0.286			
Adjusted R^2^	0.032		0.258			
ΔR^2^	0.050		0.236			
F (df1, df2)	F(12,614) = 2.704 **		F(23,603) = 10.487 ***			
ΔF (df1, df2)	—		ΔF(11,603) = 18.076 ***			
Durbin-Watson	—		2.063			

Note. HSLS = Healthy and Sustainable Lifestyle Scale; UIKE-HBS = Urinary Incontinence and Kegel Exercise Health Belief Scale; UIAAS = Urinary Incontinence Awareness and Attitude Scale. Model 1 includes 12 sociodemographic/clinical covariates. Model 2 includes 6 subscales of UIKE-HBS and 5 subscales of UIAAS (11 subscales in total) added simultaneously to Model 1. ^a^ Reference category: primary school education or lower. ^b^ Reference category: employed (active employment). ^c^ Reference category: single. * *p* < 0.05, ** *p* < 0.01, *** *p* < 0.001. In multicollinearity diagnostics, all VIF values in Model 2 remained below the accepted threshold of VIF < 10. Moderate variance inflation was observed for high school graduates (VIF = 6.709) and university graduates (VIF = 8.868), likely due to the unbalanced sample distribution across education levels; this does not threaten overall model validity and is noted in Section 5.

## Data Availability

The dataset of this study is available from the corresponding author.

## Abbreviations

UI: urinary incontinence; UIKE-HBS: the Health Belief Scale for Urinary Incontinence and Kegel Exercise; UIAAS: the Urinary Incontinence Awareness and Attitude Scale; HSLS: the Healthy and Sustainable Lifestyle Scale.
